# Withings Body Cardio Versus Gold Standards of Pulse-Wave Velocity and Body Composition

**DOI:** 10.3390/jpm10010017

**Published:** 2020-03-11

**Authors:** Scott R. Collier, Conner McCraw, Megan Campany, Austin Lubkemann, Price StClair, Hong Ji, Kathryn Sandberg, Joseph W. Morgan, Caroline J. Smith

**Affiliations:** 1 Department of Health and Exercise Science, Appalachian State University, Boone, NC 28608, USA; mccraw.conner@gmail.com (C.M.); megan.campany9@gmail.com (M.C.); A1lubkemann@gmail.com (A.L.); jpricestclair@gmail.com (P.S.); jmorgan42@gmail.com (J.W.M.); smithcj7@appstate.edu (C.J.S.); 2 Department of Medicine, Georgetown University, Washington, DC 20008, USA; jih@georgetown.edu (H.J.); sandberg@georgetown.edu (K.S.)

**Keywords:** arterial health, pulse transit time, body composition comparison, mobile health

## Abstract

Home blood pressure monitors are widely used by consumers yet cardiovascular health may be better defined by pulse-wave velocity (PWV). So far, the Withings Body Cardio scale is the only consumer device that has been designed to measure PWV and body composition, including fat mass (FM) and fat-free mass (FFM), in the home setting. While one study has demonstrated that this device meets the acceptable accuracy standards of the ARTERY Society, no study has accounted for the gravitational effect of standing on a scale on aortic-leg PWV. Purpose: The purpose of this study was to assess the accuracy of PWV and body composition as determined by the Body Cardio scale. Methods: Measurements of PWV and body composition in healthy, young males and females (*n* = 20) using the Body Cardio device were compared to PWV assessed by applanation tonometry (SphygmoCor) and body composition analysis determined by air displacement plethysmography (Bod Pod). Bland–Altman analysis and mean absolute percent error (MAPE) were used to assess accuracy. Results: Data are reported as the mean bias (95% confidence interval). The Body Cardio overestimated PWV by 0.68 m/s (−0.16, 1.51) and FM by 2.91 kg (−2.91, 8.73). Body Cardio PWV and FM estimations had a MAPE of 9.7% and 25.8%, respectively. The Body Cardio underestimated body mass (BM) and FFM by 0.11 kg (−0.41, 0.18) and 2.87 kg (−9.04, 3.30), respectively. Body Cardio BM and FFM estimations had a MAPE of 0.15% and 5.6%, respectively. Conclusions: The Body Cardio scale provides accurate measures of BM and PWV; however, it should be used cautiously for measures of FM and FFM.

## 1. Introduction

There is a growing interest from consumers, healthcare providers and researchers in using consumer devices to monitor cardiovascular health outside the healthcare provider’s office. The number of blood pressure monitors sold exceeded 235 million in 2018 in the United States alone. Pulse wave velocity (PWV) is another valuable measure of vascular health because this measure is an indicator of arterial stiffness.

Arterial stiffness relates to the mechanical properties of arteries that subsequently affect cardiovascular health. In a healthy or young population, arteries are more distensible when compared to an unhealthy or older population [1]. Arterial distensibility is critical for health because it helps attenuate pulsatile flow and aligns the timing of pressure waves returning to the heart for optimal coronary perfusion. Arterial stiffness is considered a major risk factor of cardiovascular disease and has been shown to be a predictor of cardiovascular and coronary heart events.

Another key factor in cardiovascular health is body composition. According to data from the National Health and Nutrition Examination Survey (NHANES) more than two out of three U.S. adults are considered to be overweight or obese (defined by a body mass index ≥ 25 kg·m^−2^) [2]. It is estimated that if the current rate of prevalence continues, then 86.3% of adults in the United States will be overweight or obese by the year 2030 [3]. Overweight and obesity are body weight categories defined as abnormally high or excessive fat accumulation that may impair health (Overweight & Obesity. (2018, September 17). Retrieved from https://www.cdc.gov/obesity/index.html). Excessive visceral adipose tissue is an independent risk factor for diabetes, hypertension, and all-cause mortality [4,5,6,7].

The purpose of this study was to assess the accuracy of a consumer device, the Withings Body Cardio scale (Withings, Issy-les Moulineux, France), that measures PWV and body composition including body mass (BM), fat mass (FM) and fat-free mass (FFM), as valuable measures of vascular health. Body Cardio measures of PWV and body composition were compared to laboratory gold standard measurements using the SphygmoCor Xcel (XCEL; AtCor Medical, Inc., Itasca, IL, USA) and Bod Pod (Gold, Rome, Italy).

## 2. Methods

### 2.1. Participants

Following approval of the study from the Institutional Review Board (IRB), prospective participants were contacted via face to face conversation or through an IRB approved email advertisement. All participants were required to have a personal (iOS or Android) smartphone and a service plan that they were able to use for this study. Additional inclusion criteria included being 18 years or older, healthy, and normotensive with no known cardiovascular metabolic or renal disease. Exclusion criteria included having and/or taking medication for a chronic disease (e.g., hypertension, peripheral arterial disease, effort angina, heart failure, and rheumatologic disorders). In addition, subjects were excluded if they had a health condition that limited their physical activity and/or ability to conduct the laboratory measurements. One hundred percent of the subjects interested in participating in the study met the inclusion criteria.

### 2.2. Materials and Methods

Subjects were instructed to report to the Vascular Biology and Autonomic Studies Laboratory at the Appalachian State University and be well-rested, fasted for 12 h, and without ingesting any stimulants twelve hours prior to testing. The investigator and subject reviewed an informed consent form, which resulted in the subject’s signature if they agreed to the terms and procedures of the study. Demographic data (i.e., sex, date-of-birth, race by self-report) were collected via a medical history.

Next, subjects rested while seated for fifteen minutes before brachial systolic and diastolic blood pressures were manually and automatically recorded by a medical grade stethoscope and SphygmoCor, respectively. The sphygmacor uses an arm cuff and embedded microphone to determine systolic and diastolic pressures. Manual auscultation was performed to confirm the SphygmoCor measurements. No discrepancies were observed between the stethoscope and SphygmoCor measurements.

Before all laboratory measurements, the subject was required to remove all jewelry, shoes and socks, and any excess clothing. In addition, the subject was required to wear compression clothing (e.g., spandex, compression shorts, sports bra) per standard Bod Pod procedures to eliminate negative volume displacement caused by the compressibility of air close to skin, hair, and clothing. A stadiometer and calibrated scale (Health-o-meter Professional) were used to record height and body weight. The order of instrument readings was randomly assigned. Half of the participants were measured in the Bod Pod first followed by the SphygmoCor and Body Cardio, while the reverse order was used in the other half.

#### 2.2.1. Body Cardio Assessment

The Body Cardio is a 12.9 × 12.9 × 0.8-inch, Wi-Fi-enabled consumer scale that costs USD 179.95. The scale utilizes multi-frequency bioelectric impedance analysis (BIA) technology to measure BM, FFM, and FM. Using both high and low frequencies allows for the measurement of both extracellular and intracellular fluid conductance from which an estimation of total body water (TBW) is determined. FFM can then be predicted from TWB because FM only consists of about 7% water. The Body 

Cardio uses ballistocardiography (BCG) and impedance plethysmography (IPG) to determine an aortic-leg pulse wave velocity (al-PWV). The BCG measures slight weight variations due to left-ventricle ejection of blood and corresponds well to the opening of the aortic valve. IPG measures blood volume changes at the measurement site and is used to determine the arrival time of the pulse wave to the feet from the aortic valve. Subtracting the arrival time from the left ventricle ejection of blood and the pulse wave’s arrival time at the feet results in al-PWV.

Participants were instructed to stand barefoot on the Body Cardio scale until all measures were recorded. The instrument reports measures in the following order: BM (kg), % body fat, muscle mass (kg), BM index, and PWV (m/s). The subject was instructed to stand on the Body Cardio until PWV measures were garnered. Body fat mass was calculated by multiplying the body weight by the % body fat. The Body Cardio was cleaned between subjects using 70% ethanol.

#### 2.2.2. SphygmoCor Assessment

Before all SphygmoCor (XCEL; AtCor Medical, Inc., Itasca, IL, USA) assessments, the subject was instructed to stand up next to the SphygmoCor with the Withings Body Cardio in proximal distance. The researcher measured standing brachial blood pressure after the subject had rested in the standing position for three minutes. The SphymoCor measures brachial systolic and diastolic blood pressure by inflating a cuff with a built-in pressure sensor that can measure the brachial pulsatile waveform. This brachial waveform is processed algorithmically by the SphygmoCor to determine central blood pressure, which were not used in the present study. Next, the femoral cuff was placed on the subject and carotid to femoral artery distance was measured using the fabric tape measuring device provided by SphygmoCor. Next, the researcher began assessment of PWV by placing a Doppler pen via applanation tonometry on the subject’s carotid artery. PWV was measured three times and in between measurements, body composition and PWV were determined on the Body Cardio. All subjects were within the normative hydration range in accordance with the Withings scale readout.

#### 2.2.3. Bod Pod Assessment

The Bod Pod by Cosmed is a body composition analysis system that uses air displacement plethysmography and whole-body densitometry to determine body composition (FM and FFM). Before measuring body composition with the Bod Pod, subjects were outfitted with a swim cap and instructed to breathe normally and sit still while in the Bod Pod. Thoracic gas volumes were predicted using the Bod Pod software (software version 5.4.3) for body volume corrections. Two measurements were completed over 30 min using the Bod Pod and unless the two were significantly different, i.e., more than one standard deviation, then no third measurement was made.

## 3. Statistical Analyses

An a priori power calculation was performed based on means and standard deviations from laboratory pilot data with the mean and standard deviations from the PMV (transit times in aged-matched population) and body composition data (fat and fat free mass in aged-matched populations). It was determined that 20 subjects were required to reach statistical significance with an alpha level set at 0.05 with a power of 0.93. Data was collected from each device’s particular software and the Withings’ “Healthmate” website, and compiled into a secure database. The average of three measurements were used for SphygmoCor and Body Cardio analyses. Two measurements were used for Bod Pod analyses. The data are expressed as the mean ± standard error of the mean and plotted as violin, Bland–Altman or regression graphs. Violin plots illustrate the measurement mean and the upper and lower quartiles. Additionally, a two-way ANOVA was conducted to analyze variance attributable to sex and devices. A one-sample *t*-test was performed to acquire the mean difference and 95% confidence intervals for the Bland–Altman plots. Furthermore, regression analysis was performed to assess proportional bias and to determine the Pearson correlation coefficient. The mean absolute percent error (MAPE), i.e., the error as a percentage of the overall mean, was calculated to assess the degree of error. Criterion-related validity was assessed by correlating PWV determined on the Body Cardio with measures of systolic blood pressure (SBP), heart rate (HR), BM, FM and FFM on the SphygmoCor and comparing Pearson’s r with these same correlations determined using PWV on the SphygmoCor.

## 4. Results

### 4.1. Participants

Ten male and ten female healthy adults, 18–25 years of age were included in this study. All participants were students from the University community and Caucasian. All participants completed 100% of the study. There were no sex differences in the age of the participants ((years): Men, 21.1 ± 2.0; Women, 21.5 ± 1.9) or SBP measured by the SphygmoCor ((mm Hg): Men, 112 ± 2.5; Women, 107 ± 2.0) or by manual auscultation ((mm Hg): Men, 114 ± 2.2; Women 109 ± 2.6). The men were 10 cm taller than the women (Height (cm): Men, 178 ± 1.6 vs. Women, 168 ± 1.9; *p* < 0.01). 

### 4.2. Comparison of Body Cardio PWV Measurements with SphygmoCor Determinations

**Figure 1:**
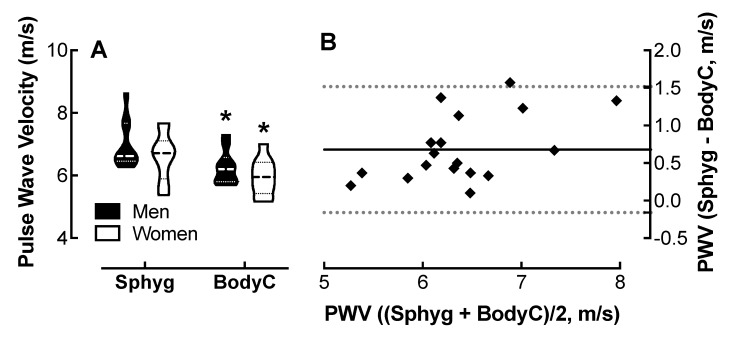
Comparison of pulse-wave velocity (PWV) measured by the Body Cardio compared with SphygmoCor determinations. (**A**) Violin plot of PWV measured by the SphygmoCor (Sphyg) and Body Cardio (BodyC) in men and women; *p* < 0.01, Sphyg vs. BodyC by two-way ANOVA; *n* = 10/group. (**B**) Q-Q plot of PWV determined by the Sphyg and BodyC. Each data point represents duplicate or triplicate measures in one study participant. PWV, pulse-wave velocity.

Analysis of variance revealed a significant effect of device on PWV measurements, though no effect of sex (Figure 1A). PWV measured on the Body Cardio was 0.7 m/s lower than that measured on the SphygmoCor.

The Bland–Altman plot (Figure 1B) shows that the Body Cardio PWV measurements follow a similar distribution to the SphygmoCor PWV determinations.

The 95% confidence interval (CI) was −0.16 to 1.5 m/s for all participants, which was similar to the CI in men and women only populations (Table 1).

The Pearson correlation coefficient (r) was 0.49 with evidence of proportional bias (*p* < 0.05). The MAPE was 9.7%.

### 4.3. Comparison of Body Cardio HR Measurements with SphygmoCor Determinations

**Figure 2:**
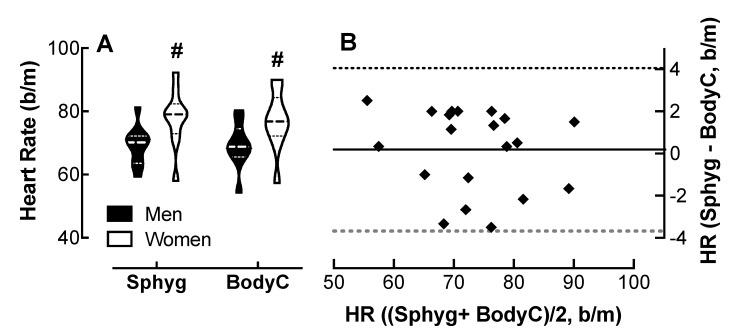
Comparison of heart rate (HR measured by the Body Cardio with SphygmoCor determinations. (**A**) Violin plot of HR measured by the Sphyg and BodyC in men and women; *p* < 0.01, male vs. female by two-way ANOVA; *n* = 10/group. (**B**) Q-Q plot of HR determined by the Sphyg and BodyC. Each data point represents duplicate or triplicate measures in one study participant. HR, heart rate.

Analysis of variance showed no effect of device on HR measurements; however, both the SphygmoCor and the Body Cardio revealed an effect of sex on HR; women had 3.8 and 8.6 b/m higher HRs, respectively, than the men (Figure 2A). The Bland–Altman plot (Figure 2B) shows that the Body Cardio HR measurements follow a similar distribution to the SphygmoCor HR determinations.

The 95% CI was −3.68 to 4.1 b/m for all participants; however, the CI was greater in the men compared to the women (Table 1).

### 4.4. Comparison of Body Cardio BM Measurements with Bod Pod Determinations

**Figure 3:**
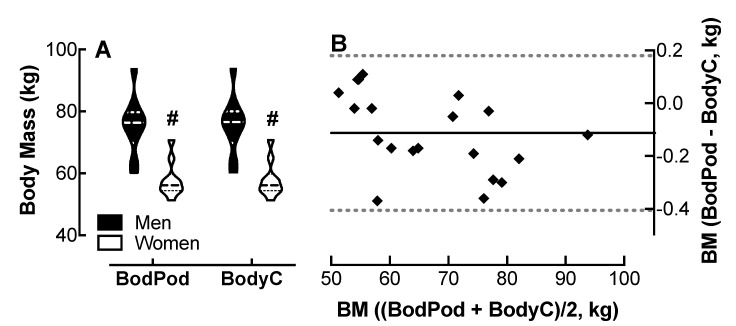
Comparison of body mass (BM) measured by the Body Cardio with Bod Pod determinations. (**A**) Violin plot of BM measured by the Bod Pod and BodyC in men and women; *p* < 0.01, male vs. female by two-way ANOVA; *n* = 10/group. (**B**) Q-Q plot of BM determined by the Bod Pod and BodyC. Each data point represents duplicate or triplicate measures in one study participant. BM, body mass.

Analysis of variance showed no effect of device on BM measurements; however, both the Bod Pod and the Body Cardio revealed an effect of sex on BM; men weighed 18 kg more than the women. (Figure 3A). The Bland–Altman plot (Figure 3B) shows the Body Cardio BM measurements follow a similar distribution to the Bod Pod determinations.

The 95% CI was −0.41 to 0.18 kg for all participants; however, the CI was greater in the women compared to the men (Table 1). The r value was 0.47 with evidence of proportional bias (*p* < 0.05). The MAPE was 0.15%.

### 4.5. Comparison of Body Cardio FM Measurements with Bod Pod Determinations

**Figure 4:**
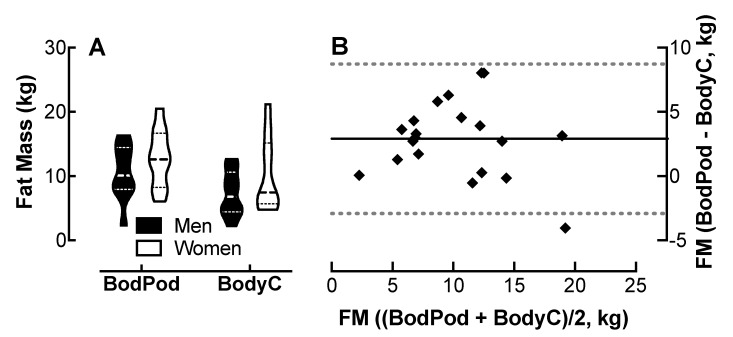
Comparison of fat mass (FM) measured by the Body Cardio with Bod Pod determinations. (**A**) Violin plot of FM measured by the Bod Pod and BodyC in men and women; *n* = 10/group. (**B**) Q-Q plot of FM determined by the Bod Pod and BodyC. Each data point represents duplicate or triplicate measures in one study participant. FM, fat mass.

Analysis of variance showed no effect of device or sex on FM measurements (Figure 4A). The Bland–Altman plot (Figure 4B) shows that the Body Cardio FM measurements follow a similar distribution to the Bod Pod determinations.

The 95% CI was −2.9 to 8.7 kg for all participants; however, the CI was greater in the women than the men (Table 1). The r value was 0.16 with no evidence of proportional bias. The MAPE was 25.8%.

### 4.6. Comparison of Body Cardio FFM Measurements with Bod Pod Determinations

**Figure 5:**
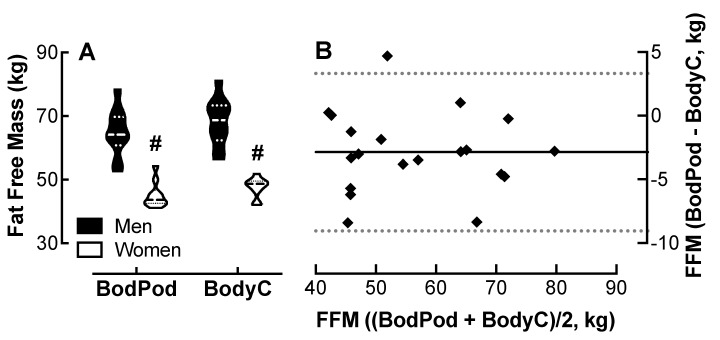
Comparison of fat free mass. (FFM) measured by the Body Cardio with Bod Pod determinations. (**A**) Violin plot of FFM measured by the Bod Pod and BodyC in men and women. *p* < 0.01, male vs. female by two-way ANOVA; *n* = 10/group. (**B**) Q-Q plot of FFM determined by the Bod Pod and BodyC. Each data point represents duplicate or triplicate measures in one study participant. FFM, fat free mass.

Analysis of variance showed no effect of device on FFM measurements, though both the Bod Pod and the Body Cardio revealed an effect of sex on FFM; men had 20 kg more FFM than the women. (Figure 5A). The Bland–Altman plot (Figure 5B) shows that the Body Cardio FFM measurements follow a similar distribution to the Bod Pod determinations.

The 95% CI was −9.0 to 3.3 kg for all participants; however, the CI was greater in the women than the men (Table 1). The r value was 0.06 with no evidence of proportional bias. The MAPE was 5.6%.

### 4.7. Relationship of SBP Measured on the SphygmoCor with PWV as a Function of Device

**Figure 6:**
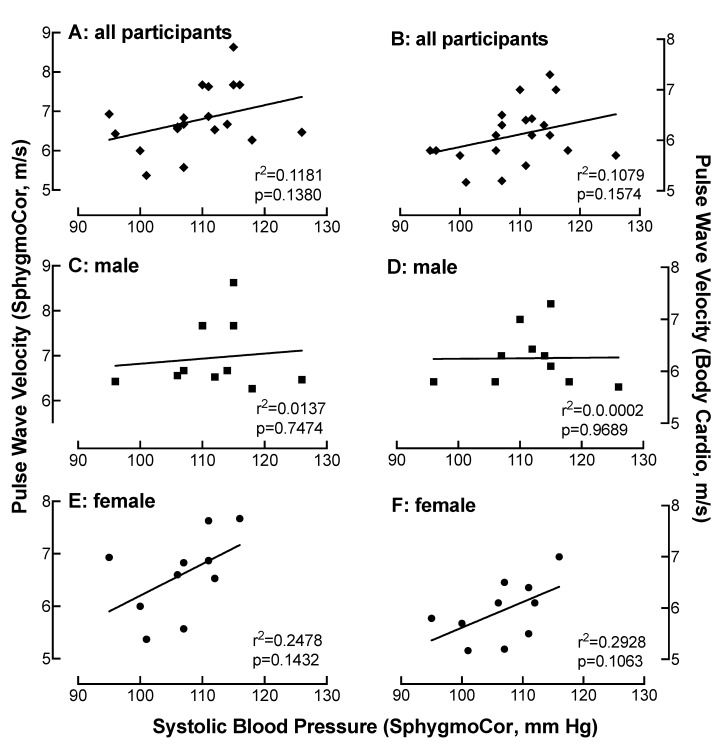
Relationship of systolic blood pressure (SBP) measured on the SphygmoCor with PWV as a function of device. Comparison of SBP with PWV measured on the SphygmoCor (**A**,**C**,**E**) or the Body Cardio (**B**,**D**,**F**) in all participants (**diamond**) (**A**,**B**) or specifically in the male (**square**) (**C**,**D**) or female (**circle**) (**E**,**F**) population. Each data point represents duplicate or triplicate measures in one study participant. The confidence interval was greater in the women compared to the men (Table 1).

There was a weak correlation between PWV and SBP measured on the SphygmoCor in all participants (r < 0.34) (Figure 6A). There was a stronger correlation between PWV and SBP (r = 0.50) in the female only population (Figure 6C), while little correlation was observed in men (r < 0.2) (Figure 6E). Similarly, when PWV was measured on the Body Cardio, there was a weak correlation between PWV and SBP in all participants and in the women (r = 0.29) (Figure 6D), which was not present in the men (r < 0.2) (Figure 6F).

### 4.8. Relationship of HR Measured on the SphygmoCor with PWV as a Function of Device

There was a small relationship between PWV and HR measured on the SphygmoCor in all participants (r < 0.2) or specifically in the male (r < 0.2) or female (r < 0.2) population. Similarly, there was a slight to weak relationship between HR measured on the SphygmoCor with PWV measured on the Body Cardio in all participants (r < 0.2) or in the male (r = 0.25) and female (r = 0.31) only populations.

### 4.9. Relationship of BM Measured on the Bod Pod with PWV as a Function of Device

**Figure 7:**
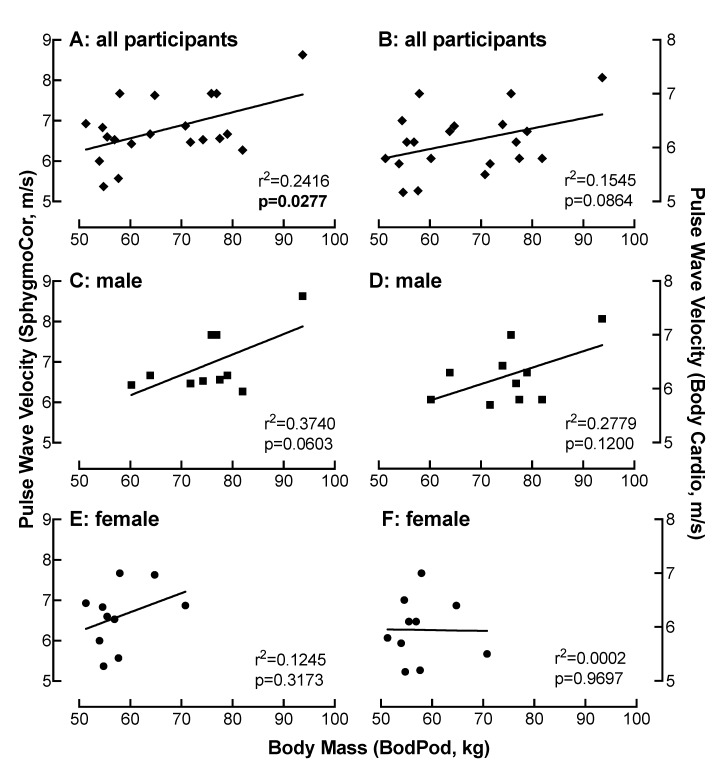
Relationship of BM measured on the Bod Pod with PWV as a function of device. Comparison of BM with PWV measured on the SphygmoCor (**A**,**C**,**E**) or the Body Cardio (**B**,**D**,**F**) in all participants (**diamond**) (**A**,**B**) or specifically in the male (**square**) (**C**,**D**) or female (**circle**) (**E**,**F**) population. Each data point represents duplicate or triplicate measures in one study participant.

There was a moderate correlation between PWV measured on the SphygmoCor with BM determined by the Bod Pod in all participants (r = 0.49) (Figure 7A). This correlation was strong in men (r = 0.61) (Figure 7C) and weak in women (r < 0.2) (Figure 7E). These correlations between PWV and BM were similar when measured on the Body Cardio in all participants (r = 0.39) (Figure 7B) or in the male (r = 0.53) (Figure 7D) and female (r < 0.2) (Figure 7F) only populations.

### 4.10. Relationship of FM Measured on the Bod Pod with PWV as a Function of Device

**Figure 8:**
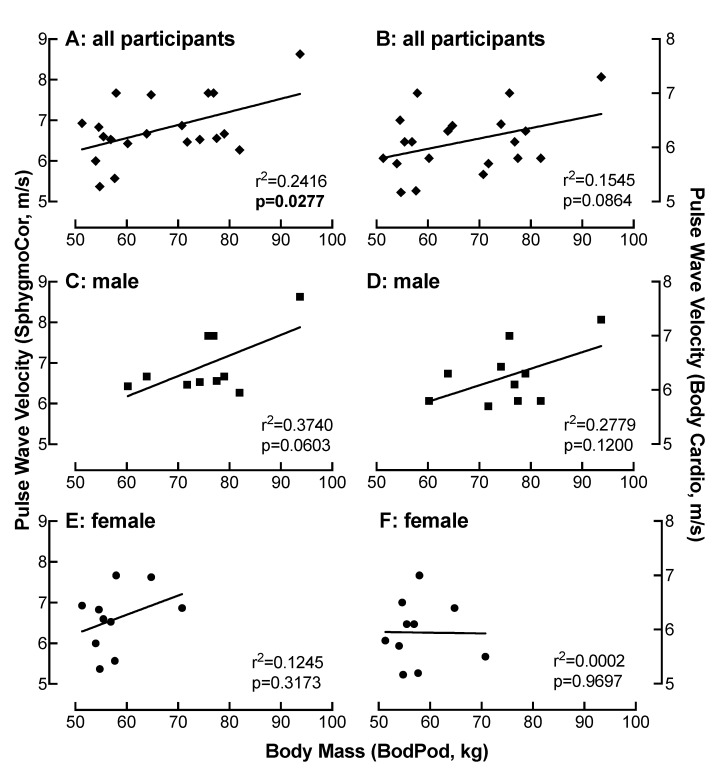
Relationship of FM measured on the Bod Pod with PWV as a function of device. Comparison of FM with PWV measured on the SphygmoCor (**A**,**C**,**E**) or the Body Cardio (**B**,**D**,**F**) in all participants (**diamond**) (**A**,**B**) or specifically in the male (**square**) (**C**,**D**) or female (**circle**) (**E**,**F**) population. Each data point represents duplicate or triplicate measures in one study participant.

There was little relationship between PWV measured on the SphygmoCor with FM determined on the Bod Pod in all participants (r < 0.2) (Figure 8A); however, in the male only population, there was a moderate correlation between PWV and FM (r = 0.52) (Figure 8C), which was absent in women r < 0.2) (Figure 8E). Similarly, when PWV was measured by the Body Cardio, there was a weak correlation with FM in the male only population (r = 0.30) (Figure 8D) whereas there was little relationship observed in all participants (r < 0.2) (Figure 8B) or in the female only population (r < 0.2) (Figure 8F).

### 4.11. Relationship of FFM Measured on the Bod Pod with PWV as a Function of Device

**Figure 9:**
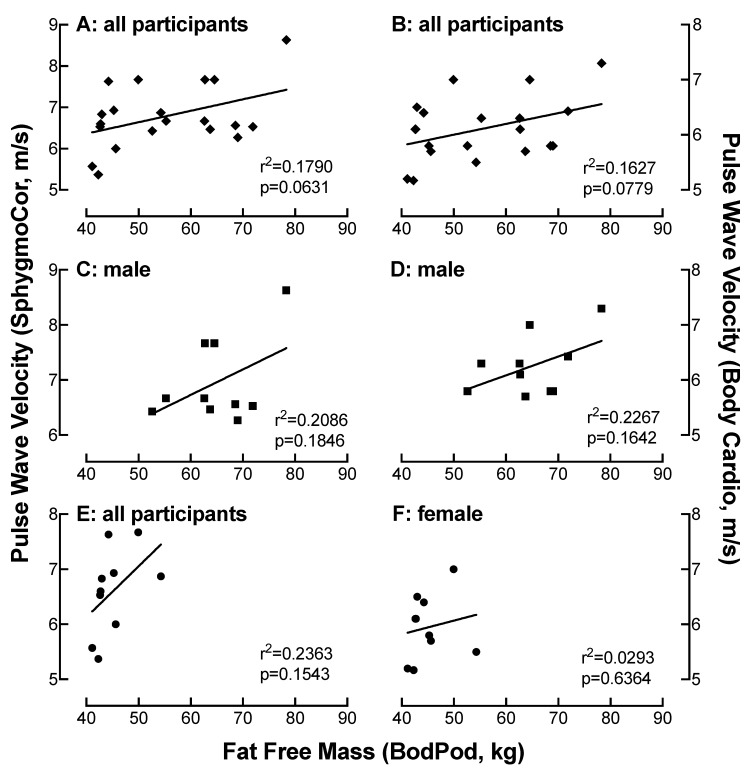
Relationship of FFM measured on the Bod Pod with PWV as a function of device. Comparison of FFM with PWV measured on the SphygmoCor (**A**,**C**,**E**) or the Body Cardio (**B**,**D**,**F**) in all participants (**diamond**) (**A**,**B**) or specifically in the male (**square**) (**C**,**D**) or female (**circle**) (**E**,**F**) population. Each data point represents duplicate or triplicate measures in one study participant.

There was a moderate correlation between PWV measured on the SphygmoCor with FFM determined on the Bod Pod in all participants (r = 0.42) (Figure 9A) and in the male (r = 0.46) (Figure 9C) and female (r = 0.49) (Figure 9E) only populations. Similar associations were observed when PWV was measured by the Body Cardio in all participants (r = 0.40) (Figure 9B) and in the male population (r = 0.48) (Figure 9D) but not in the female only group (r < 0.2) (Figure 9F).

## 5. Discussion

The major finding of this study is that the Withings Body Cardio scale accurately assesses PWV. The mean difference between Body Cardio and SphygmoCor PWV determinations was less than 0.2 with a SD of 0.57 (m/s). This difference in PWV measures between devices is well within the accuracy standards deemed acceptable by the ARTERY Society, that is, less than 1.0 m/s and less than 1.5 m/s SD [8].

These findings extend a previous report, where Butlin et al. [9] found similar agreement of PWV measures between the Body Cardio with the SphygmoCor but relied on self-reported height and did not account for the gravitational effects of standing upright on PWV [9]. We determined height by a stadiometer and measured PWV using both devices while standing. Gravity is known to affect PWV, however, there are few studies that elucidate this phenomenon. Schroeder et al. (2017) demonstrated that the more upright position one’s body is, the greater the arterial stiffness within that physiological system [10]. Further, they found that the change in position is independent of blood pressure. This was demonstrated when one went from a supine to a seated position. The increases shown in arterial stiffness were only found to be dependent on arterial pressure and not related to any other physiological influences. The arterial tree is heterogeneous. Elastic properties diminish from the central to peripheral arteries, thereby creating an increase in amplitude of the pressure wave, known as pressure amplification. Frank, Bramwell, and Hill derived this propagative model of the circulatory system in the early 20th century. They concluded that pressure amplification and the PWV is inversely related to the distensibility. This principal led to a theoretical model that is the basis of current technology used in PWV measurements [11].

These seminal studies subsequently led an expert consensus group to establish the carotid-to-femoral PWV (cf-PWV) as the gold standard for non-invasive measurement of arterial stiffness [11,12,13]. Multiple meta-analyses have revealed that cf-PWV measures improve the prediction of cardiovascular events and mortality, independently of standard risk factors like hypertension, dyslipidemia, or high blood glucose [14,15,16].

The SphygmoCor XCEL uses applanation tonometry for high-frequency arterial wave form acquisition and volumetric displacement within a cuff for femoral pulse. The cf-PWV is calculated by measuring the transit time between carotid and femoral pulse and dividing it by the pulse wave’s travel distance. This travel distance is estimated by measurement of several surface points along the body to most accurately determine carotid-femoral length [14]. The SphygmoCor devices by AtCor Medical are considered a gold standard of reference for determining PWV. They have been validated against the ARTERY Society’s PWV guidelines [1,17] and their use is cited over 1000 times in peer-reviewed research articles. The Body Cardio uses ballistocardiography and impedance plethysmography to determine al-PWV [18]. Ballistocardiography measures slight weight variations due to left-ventricle ejection of blood and corresponds well to the opening of the aortic valve [18]. Impedance plethysmography measures blood volume changes at the measurement site and is used in the consumer scale to determine the arrival time of the pulse wave to the feet from the aortic valve. Subtracting the arrival time from the left ventricle ejection of blood and the pulse wave’s arrival time at the feet enable determination of al-PWV. Measurement of PWV is typically made when the body is supine. In a standing position, gravity causes an increase in blood volume in the capacitance vessels of the legs, which results in a fall of central blood volume and preload. Consequently, this leads to a decrease in mean arterial pressure. A baroreflex-mediated compensatory increase in peripheral resistance and heart rate then stabilizes the mean arterial pressure in healthy individuals within three minutes. Measuring PWV while standing may be limited by the contractions of stabilization muscles in the legs causing increased venous return by the skeletal muscle pump. Contrarily, subjects with orthostatic hypotension, hypertension, or other cohorts could change the results of the present study and this warrants further research.

The Body Cardio scale is a more convenient and a less expensive alternative to devices like the SphygmoCor for measuring arterial stiffness. Furthermore, it can be used repeatedly outside the healthcare provider’s office. An affordable, portable, and accurate scale for measurement of PWV that can be monitored over time has implications for consumers, healthcare providers and researchers [19]. There has been an explosion in the interest of consumers in using PWV to follow their health. Thus, the Body Cardio has the potential to motivate consumers to make healthy lifestyle choices. The ability to repeatedly assess PWV at home is of interest to healthcare providers because this measure of arterial stiffness provides additional information that is complimentary to other assessments of a patient’s overall cardiovascular health and it improves prediction of cardiovascular disease events and mortality independently of lifestyle and standard risk factors. Furthermore, measuring PWV at home minimizes false readings at the healthcare provider’s site due to patient anxiety. The ability to record multiple measures of PWV over time in a non-clinical setting for a fraction of the cost of conducting the same measures using the SphygmoCor promotes longitudinal and large cohort studies of PWV by researchers. The second major finding of this study is that the none of the Body Cardio measures of body composition other than BM meet guidelines of acceptability. These include guidelines proposed in a study that compared body composition measurements made by dual-energy X-ray absorptiometry to the Bod Pod [20]. FM and FFM measures were deemed acceptable if the percent error was less than 1.5%.

The Body Cardio utilizes multi-frequency BIA technology to measure BM, FM, and FFM. The scale also wirelessly connects to a smart phone application where changes in body composition and weight can be tracked over time. While regular self-weighing and the use of smartphone health applications may lead to improved health outcomes, the Body Cardio has not been compared to gold standard measurement methods of body composition nor validated by an independent laboratory [21,22].

Bioimpedance technology has progressed over the last decade. However, recent study showed that when it was compared to dual-energy X-ray absorptiometry, the percent error between methods was nearly 9% [23]. A principle the BIA uses is that of a cylinder’s volume where volume equals length multiplied by the cross-sectional area and impedance is inversely proportional to its cross-sectional area. This principle also assumes that the cylinder’s material is homogeneously conductive. The human body, however, is not cylindrical, and tissues vary in conductivity [24,25]. To address this issue, engineers developed a multi-frequency BIA.

Using both high and low frequencies allows the electrical current to penetrate the cell membrane and consequentially, account for both the extracellular fluid and intracellular fluid conductance from which an estimation of total body water is determined. FFM is predicted from total body water because only about 7% of FM consists of water [24,25]. Adipocytes vary in number and size. Thus, the amount of water within FM varies, which leads to inherent error in any extrapolations based on this assumption. Furthermore, the deposition of insulated tissues within the body including adipose tissue impacts the impedance of surrounding conductive tissues according to the mixing theory [26]. We compared body composition analysis using the Body Cardio with determinations made by the Bod Pod, which is a device that utilizes air displacement plethysmography.

This system uses a measuring chamber of a known pressure and volume to calculate the volume of the subject. Poisson’s Law, the pressure-volume relationship at a constant temperature and humidity, and adjustments for thoracic lung volume, are used to measure the volumetric displacement the subject imposes on the chamber [27]. Body density is then derived using other demographic measures like height and BM in addition to body volume. Like hydro-densitometry, body density is then used to estimate a two-compartment body composition model consisting of FM and FFM. The Bod Pod has been validated when compared to DXA and for test-retest reliability [28,29]. Despite its validity in the measurement of body composition, the Bod Pod is not commonly available for consumers because the procedure is often costly and takes time to complete. We expected to detect sex differences in the relationship between PWV and body composition because sexual dimorphism exists in fat distribution, which affects cardio-metabolic disease risk. Males typically have an android phenotype characterized by a greater accumulation of visceral fat [30]. Females generally have a gynoid phenotype with greater subcutaneous adipose tissue accrual around the gluteo-femoral region than visceral/abdominal adipose deposition. Increased lipoprotein lipase activity in the visceral region and its suppression by testosterone in the femoral region of men likely contribute to these sex differences in fat deposition [31,32]. Growth of fat mass can occur either by growth in volume of pre-existing adipocytes (i.e., hypertrophy) or through recruitment of new preadipocytes (i.e., hyperplasia) [33]. Studies in rodents suggest that the subcutaneous depot increases by adipocyte hyperplasia while visceral fat accumulates by adipocyte hypertrophy [34]. These sex differences in adipose tissue expansion and adipocyte deposition could lead to sex differences in body composition analysis based on bioelectrical impedance.

The limitations of this study include the small sample size, which could have obscured sex differences in PWV measures by the Body Cardio device. Other study limitations include the young age of the cohort, they were all Caucasian and their healthy status. Thus, the accuracy of PWV measures and the relationships observed between PWV, blood pressure and body mass may be specific to this study population and may not represent individuals at higher risk for developing cardiovascular disease such as older individuals and those with hypertension [35]. However, this age category is one of the largest consumers of this technology. Limitations associated with using GWP like the Body Cardio scale for research purposes include changes in company ownership or software updates in the midst of a study, which can jeopardize the ability to compare measures within the study. Companies could promote the use of these devices for healthcare and research by improving the dialogue between consumers and device manufactures regarding device accuracy.

In conclusion, the Withings’ Body Cardio scale has the potential to improve the arterial health of consumers by providing accurate assessment of PWV in the home. This information can serve to motivate individuals to make healthy life style choices as well as offer health care providers the ability to monitor their patient’s health. Furthermore, this feature offers researchers an inexpensive method for studying PWV longitudinally in an ecological setting. In contrast, the use of the Body Cardio to assess percent of body fat must be used with caution as the MAPE is greater than 25%.

## Figures and Tables

**Table 1:** Altman statistical analyses.

Parameter	Cohort	Bias ± SD	95% Limits of Agreement
**PWV**	Men + Women	0.68 ± 0.43	−0.16 to 1.50
	Men only	0.71 ± 0.44	−0.17 to 1.58
	Women only	0.65 ± 0.43	−0.19 to 1.50
**HR**	Men + Women	0.18 ± 1.97	−3.68 to 4.05
	Men only	−0.05 ± 2.37	−4.70 to 4.60
	Women only	0.42 ± 1.57	−2.65 to 3.49
**BM**	Men + Women	−0.11 ± 0.15	−0.41 to 0.18
	Men only	−0.18 ± 0.12	−0.42 to 0.05
	Women only	−1.04 ± 0.15	−0.34 to 0.25
**FM**	Men + Women	2.91 ± 2.97	−2.91 to 8.73
	Men only	3.28 ± 2.39	−1.41 to 7.97
	Women only	2.54 ± 3.55	−4.42 to 9.50
**FFM**	Men + Women	−2.87 ± 3.15	−9.05 to 3.31
	Men only	−3.26 ± 2.55	−8.25 to 1.74
	Women only	−2.48 ± 3.76	−9.86 to 4.89
